# Solitary rectal ulcer transformation to cap polyposis in a 15-year-old child

**DOI:** 10.1186/s12876-022-02122-2

**Published:** 2022-03-07

**Authors:** Abdolreza Emami, Javad Shokri Shirvani, Akramasadat Hosseini, Seyed Hossein Hamidi

**Affiliations:** 1 Student Research Committee, School of Medicine, Babol University of Medical Sciences, Babol, Islamic Republic of Iran; 2 Department of Internal Medicine, School of Medicine, Babol University of Medical Sciences, GanjAfrooz Street, 47176-47745 Babol, Mazandaran Islamic Republic of Iran; 3 Department of Pathology, School of Medicine, Babol University of Medical Sciences, Babol, Islamic Republic of Iran; 4 Department of Anesthesiology, School of Medicine, Babol University of Medical Sciences, Babol, Islamic Republic of Iran

**Keywords:** Adolescent, Rectum, Ulcer, Colonic polyps, Granulation tissue

## Abstract

**Background:**

Cap polyposis (CP) is a benign, non-malignant inflammatory disease that affects the rectum. It usually occurs during the 5th decade of life, but children could also be affected. Its specific pathology is unknown. Due to the clinical, endoscopic, and histologic similarities with other disorders such as inflammatory bowel disease, a thorough histologic evaluation is critical to avoid unnecessary interventions. This study presents a 15-year-old child with a previously reported case of solitary rectal ulcer (SRU) that developed into CP determined by colonoscopy and histologic findings.

**Case presentation:**

A 15-year-old boy who was previously diagnosed with SRU presented to our office with rectal bleeding, mucoid discharge, and abdominal pain. Additional colonoscopy evaluation revealed multiple polyposes varying in size and shape limited to the rectum. Histologic examination revealed a characteristic cap of granulation tissue covering tortuous nondysplastic crypts in the inflamed stroma, indicating that SRU had transformed into CP. Based on the assessments, we planned to perform endoscopic mucosal resection of the lesions in multiple sessions.

**Conclusions:**

Despite the rarity of CP, the transformation from SRU may be one of its etiologies. Thus, thorough serial histologic evaluation is critical in children with rectal bleeding to avoid unnecessary or harmful interventions.

## Background

Cap polyposis (CP) is a rare benign inflammatory condition characterized by colorectal polyps with a fibrinopurulent mucoid cap [1]. Due to its rarity, the incidence rate is unknown, but most reported cases are men or women in their fifth decade of life, and there have been fewer than 100 reported cases of children affected by this disorder worldwide [2].

Rectal bleeding and mucoid discharge are the most frequently reported symptoms [1], prompting colonoscopy to rule out other possibilities such as inflammatory bowel disease (IBD), malignancies, and familial adenomatous polyposis (FAP) [3]. During colonoscopy evaluation, single or multiple polyps varying in size and shape in the rectum or colon may be observed [2], raising the possibility of cap polyposis. A definitive diagnosis requires the collection of numerous tissue samples in order to confirm the disease histologically [4]. We present a case of solitary rectal ulcer (SRU) in a 15-year-old boy that developed into CP over time. He complained of worsening symptoms associated with his initial diagnosis of SRU, including rectal bleeding, constipation, and mucoid discharge. Colonoscopy revealed numerous polyps of various sizes and shapes throughout the rectum, and histopathologic examination confirmed the diagnosis.

## Case presentation

**Fig. 1:**
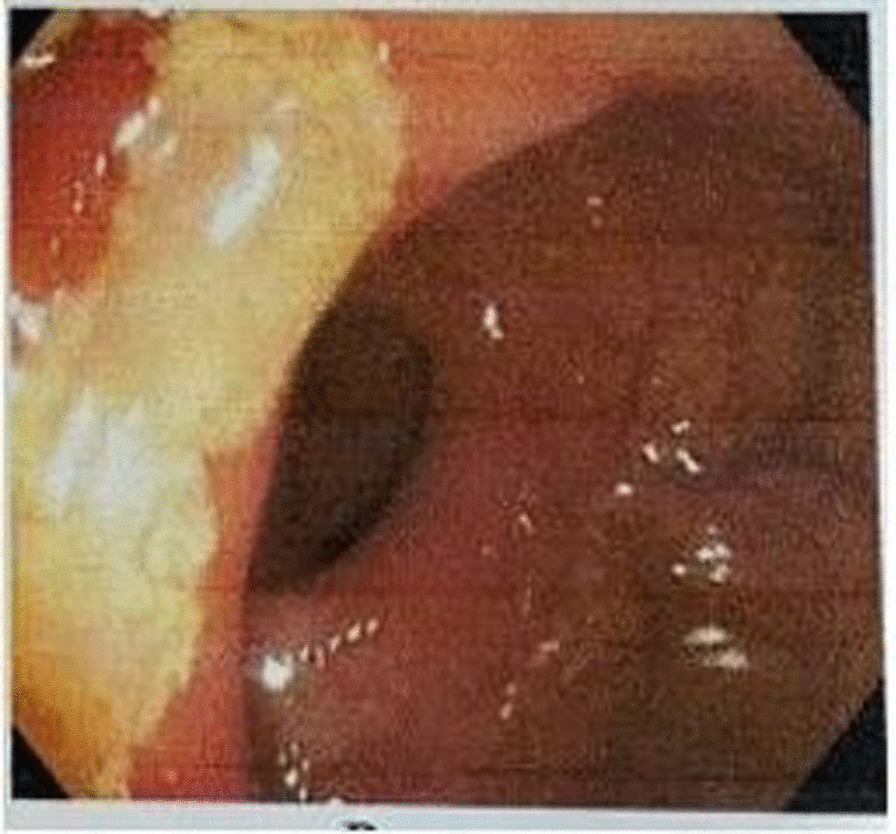
The patient’s first colonoscopy evaluation four years ago, revealing no abnormalities except for a solitary ulcer in the rectum

A 15-year-old boy who was previously diagnosed with SRU presented to our office with frequent bloody stool, chronic abdominal pain, constipation, and mucoid discharge. Five years earlier, his symptoms began with intermittent vague abdominal pain and bloody stool. Progressive deterioration of symptoms includes increased blood in the stool, new onset of mucoid discharge, and new onset of constipation. He complained of straining during defecation, difficulty passing hard and narrowed stool, and a sense of incomplete defecation that typically required manual finger evacuation. There was no previous history of surgery, prescription medication, or any other medical condition. He experienced no diarrhea, food intolerance, or significant weight loss. Additionally, his family history was negative for gastrointestinal disorders, including malignancies, IBD, and FAP. He had his first colonoscopy approximately four years prior, which revealed a solitary ulcer in the rectum (Fig. 1).

The initial histologic examination revealed regenerative crypts in the lamina propria surrounded by fibrosis and smooth muscle proliferation, confirming the diagnosis of SRU. A previous laboratory evaluation revealed a WBC of 8100 × 10^9^ cells/liter, Hb of 13.2 g/dL, MCV of 79.8 FL, platelets of 251,000 × 10^10^/unit, FBS of 85 mg/dL, TSH of 3.49 mIU/liter, AST of 12 units/liter, ALT of 16 units/liter, ALP of 391 units/liter, and ferritin of 22.2 mcg/liter, stool exam negative for occult blood, ova of parasites, and protozoa cyst.

On physical examination, his vital signs were stable, and there were no abnormal findings in his head, neck, chest, or abdomen. A perineal examination revealed no skin tags, fissures, visible prolapse, or other indications of child abuse. However, multiple irregular mass-like lesions were detected during the digital rectal examination.

**Fig. 2:**
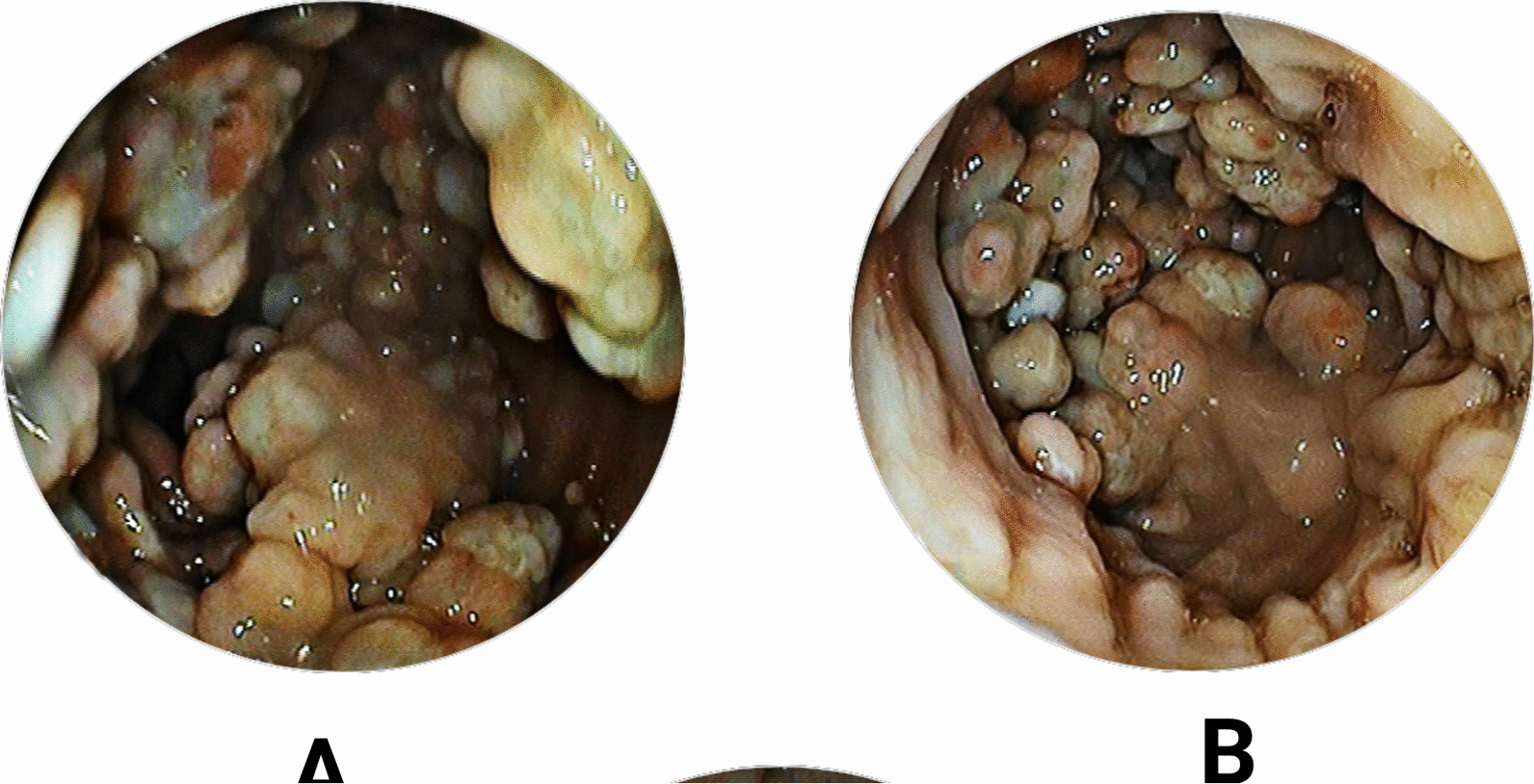
**A** Multiple diffuse polyposes varying in shapes and size ranging from 5 to 15 mm, extending from the middle section of the rectum to the rectosigmoid junction. Other sections of the colon were normal. **B** Normal appearance of the anal verge

We performed a colonoscopy, revealing multiple diffuse polyposes ranging in shape and size from 5 to 15 mm, extending from the rectum’s middle section to the rectosigmoid junction (Fig. 2).

**Fig. 3:**
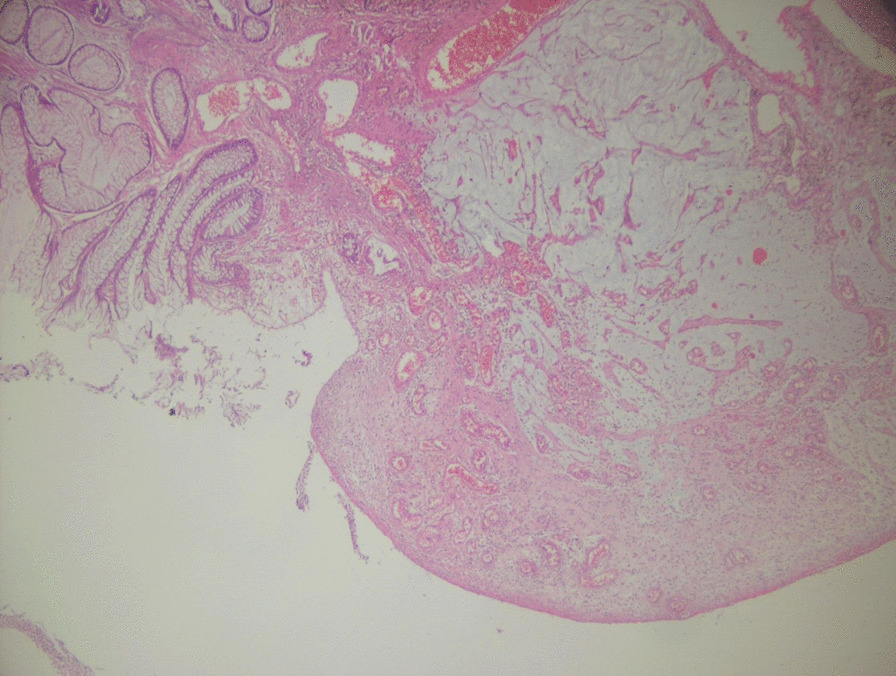
Microscopical examination at low magnification. Structure of a polypoid with an ulcerated granulation tissue cap and inflammatory exudates on the surface

**Fig. 4:**
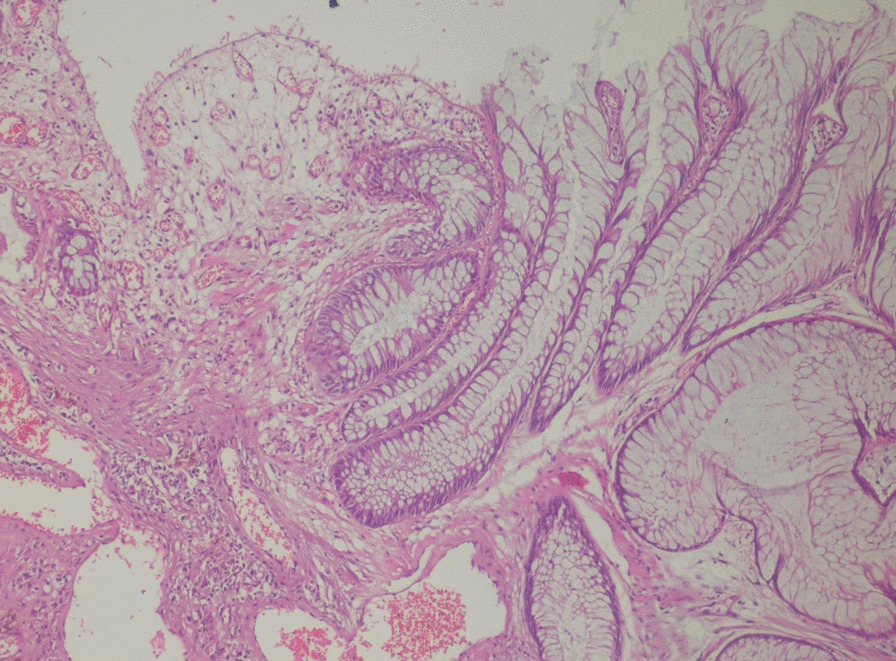
At a higher magnification, granulation tissue is visible overlying tortuous crypts with basally located nuclei, as well as the presence of smooth muscle fibers and congested vessels in the inflamed stroma

Multiple biopsies were taken and sent for histopathologic evaluation, which revealed a polypoid structure with a cap of ulcerated granulation tissue, inflammatory exudates on the surface, and tortuous non-dysplastic crypts with mucin spillage in the inflamed stroma, which included smooth muscle fibers and congested vessels (Figs. 3 and 4). Thus, the diagnosis of CP was confirmed based on these new findings.

To rule out protein-losing enteropathy, total serum protein and albumin concentrations were determined to be normal (6.7 and 4.3 g/dL, respectively).

Until histopathologic confirmation of CP is obtained, conservative measures such as a high-fiber diet, the use of laxative agents, and avoidance of manual stool evacuation are recommended to the patient. After histopathologic confirmation, we decided to perform endoscopic mucosal resection (EMR) and resected as many polyps as possible during the first session, considering the severity of the symptoms, multiple polyps in the rectum, and the lack of a standard established treatment plan. During the patient’s 3-month follow-up, the patient underwent two additional colonoscopy sessions, during which we removed the majority of the remaining polyps using EMR, but complete eradication was not possible. Nonetheless, the patient’s symptoms improved, the rectal bleeding stopped, and the abdominal pain subsided.

## Discussion and conclusion

As previously stated, CP, even in adults, is considered a rare disease, with fewer than 100 cases reported until 2017 [2]. They are most frequently reported in patients in their fifth decade of life, with a slight predominance in women aged 12 to 76 years [5]. However, children may also be impacted. Evidently, the youngest child reported was 11 months old [6], and another study by Li et al. found that the affected child’s median age was 13 years [4]. Although the exact pathophysiology of CP is unknown, inflammation, infection, particularly Helicobacter pylori, and abnormal intestinal motility have all been suggested as possible mechanisms, primarily due to the disease’s similarities to other diseases such as IBD [2, 4].

Rectal bleeding was the most frequently reported clinical manifestation in children, followed by mucoid discharge, constipation, diarrhea, and abdominal pain. Tenesmus, straining during defecation, the sensation of incomplete defecation, the need for manual stool evacuation, weight loss, and protein-losing enteropathy have all been reported in various studies at differing prevalence rates [2, 4]. Additionally, our patient presented with rectal bleeding, mucoid discharge, abdominal pain, and a five-year history of straining during defecation, progressing in severity over time. Although anemia, changes in serum inflammatory markers, and a decrease in total serum protein or albumin levels have been reported in some cases [4], our patient had no laboratory abnormalities.

Almost all lesions found during colonoscopy evaluation of adults or children are located in the rectum, occasionally extending to the anal canal, and less frequently in the sigmoid colon [2]. However, Stomach involvement is reported in the adult-only [7]. Our patient’s involvement was restricted to the rectum, with no extension to the proximal or distal sections. Sessile, pedunculated, ulcerated, or flat lesions are all possible presentations of lesions of varying sizes and numbers [2, 4].

As mentioned previously, our patient had multiple polyposis lesions ranging in size from 5 to 15 mm. CP is characterized histopathologically by hyperplastic crypts that are attenuated toward the ulcerated mucosal surface and covered in a thick layer of fibrinopurulent exudate. However, there are some similarities with other diseases such as IBD and mucosal prolapse syndrome, which results in frequent misdiagnosis and unnecessary or harmful evaluations or therapeutic strategies [2, 4].

Our patient was initially diagnosed with SRU following a colonoscopy and histological examination. However, our assessment revealed significantly different findings, possibly due to misdiagnosis or a possible mechanism of SRU to CP transformation.

Due to the rarity, unknown etiology, and uncertainty surrounding diagnostic criteria for CP, treatment strategies vary across studies. These include conservative measures such as consuming a high-fiber diet, bowel training, and avoiding manual stool evacuation and constipation in patients with evidence of infection, such as a positive breath urea test for Helicobacter pylori, aminosalicylates either topical or systemic, steroids, and TNF-alpha based on the disease’s possible inflammatory process. Moreover, alternative measures including endoscopic procedures such as EMR and polypectomy or even surgery such as rectopexy or total proctosigmoidectomy may be used in patients who are resistant to other treatments or who experience recurrence of symptoms interfering with daily activities [2, 8, 9].

In our case, we decided to perform polypectomy in multiple sessions due to the patient’s persistent symptoms, insufficient response to conservative measures, and endoscopic findings of multiple and large polyps, which may exacerbate symptoms over time.

Despite the rarity of CP, the transformation from SRU may be a possible etiology in children who present with rectal bleeding, mucoid discharge in the stool, or other gastrointestinal symptoms that overlap with IBD, FAP, or malignancies. As a result of the benign nature of CP, a thorough colonoscopy and histology evaluation are required to establish a definitive diagnosis and avoid proceeding with extreme or harmful treatment measures. As was the case with our patient, we recommend repeating histopathology evaluation in the event of the progression of symptoms or lack of response to previous treatments and considering SRU transformation to CP. Numerous treatment strategies have been proposed, ranging from conservative measures to surgery. However, no universally accepted treatment has been established. Additional research is required to understand the mechanism better, develop diagnostic criteria, and ultimately develop effective therapies.

## Data Availability

Data sharing is not applicable to this article as no datasets were generated or analyzed during the current study.

## Abbreviations

CP: Cap polyposis
SRU: Solitary rectal ulcer
IBD: Inflammatory bowel disease
FAP: Familial adenomatous polyposis
TSH: Thyroid-stimulating hormone
AST: Aspartate aminotransferase
ALT: Alanine aminotransferase
ALP: Alkaline phosphatase
EMR: Endoscopic mucosal resection
WBC: White blood cell
Hb: Hemoglobin
g/dL: Gram per deciliter
MCV: Mean corpuscular volume
FL: Femtoliter
mg/dL: Milligram per deciliter
mIU/liter: Milli-international unit per liter
mcg/liter: Microgram per liter
